# Supplementary material for: Origin and role of the cerebrospinal fluid bidirectional flow in the central canal
Source: eLife. 2020 Jan 9;9:e47699. doi: 10.7554/eLife.47699 (PMC6989091; doi:10.7554/eLife.47699)
Supplement: Transparent reporting form [file elife-47699-transrepform.docx]

***eLife’s* transparent reporting form**

We encourage authors to provide detailed information *within their submission* to facilitate the interpretation and replication of experiments. Authors can upload supporting documentation to indicate the use of appropriate reporting guidelines for health-related research (see [EQUATOR Network](http://www.equator-network.org/%20)), life science research (see the [BioSharing Information Resource](https://biosharing.org/)), or the [ARRIVE guidelines](http://www.plosbiology.org/article/info:doi/10.1371/journal.pbio.1000412) for reporting work involving animal research. Where applicable, authors should refer to any relevant reporting standards documents in this form.

If you have any questions, please consult our Journal Policies and/or contact us: [editorial@elifesciences.org](mailto:editorial@elifesciences.org).

**Sample-size estimation**

- You should state whether an appropriate sample size was computed when the study was being designed
- You should state the statistical method of sample size computation and any required assumptions
- If no explicit power analysis was used, you should describe how you decided what sample (replicate) size (number) to use

Please outline where this information can be found within the submission (e.g., sections or figure legends), or explain why this information doesn’t apply to your submission:

The CSF flow profiles, as well as the diameter and cilia measurements were obtained from different experiments on very large Ns (>100 fish embryos, >10 clutches thousands of cilia), and appear robust. We could verify the statistical robustness by computing the histograms on random sub-populations and verify that the distributions have similar characteristics.

The description of the three canals connecting the brain ventricles to the central canal were shown in supplemental movies on a few embryos only, but these canals were found in all > 20 fish embryos we have tested throughout hours of imaging sessions.

The ablation experiment in Figure 8 was performed on ~15 embryos in each group (ablated & control siblings) from the same clutch raised at low density to ensure synchronization of their development. This is a very challenging experiment and time-consuming experiment, and we therefore performed them with extra care but limited our Ns to 15 fish per group.

**Replicates**

- You should report how often each experiment was performed
- You should include a definition of biological versus technical replication
- The data obtained should be provided and sufficient information should be provided to indicate the number of independent biological and/or technical replicates
- If you encountered any outliers, you should describe how these were handled
- Criteria for exclusion/inclusion of data should be clearly stated
- High-throughput sequence data should be uploaded before submission, with a private link for reviewers provided (these are available from both GEO and ArrayExpress)

Please outline where this information can be found within the submission (e.g., sections or figure legends), or explain why this information doesn’t apply to your submission:

No outliers were ever excluded and the data have been entirely kept for plots and statistical analysis.

For each experiment presented in the manuscript, few trials were performed prior to acquiring large amount of data to optimize our protocol and ensure the technical replicability.

Once the protocol was set, biological replicates were accumulated for all experiments. The CSF flow and central canal diameter measurements were performed on several clutches (up to 17 for the CSF flow measurements).

Figure 4C compares WT and mutant embryos from the same clutch, and we observed the same effect on all clutches imaged from incross of mutants with defective cilia.

**Statistical reporting**

- Statistical analysis methods should be described and justified
- Raw data should be presented in figures whenever informative to do so (typically when N per group is less than 10)
- For each experiment, you should identify the statistical tests used, exact values of N, definitions of center, methods of multiple test correction, and dispersion and precision measures (e.g., mean, median, SD, SEM, confidence intervals; and, for the major substantive results, a measure of effect size (e.g., Pearson's r, Cohen's d)
- Report exact p-values wherever possible alongside the summary statistics and 95% confidence intervals. These should be reported for all key questions and not only when the p-value is less than 0.05.

Please outline where this information can be found within the submission (e.g., sections or figure legends), or explain why this information doesn’t apply to your submission:

The Numbers and p-values are reported in the figure legends. Figures show means and standard deviations, except from the flow profiles that show the standard error of the mean (of the distribution of beads velocity), since we were interested in the accuracy of the flow velocity rather than the spread of the distribution. This is particularly critical here, as Brownian motion significantly broadens the speed distribution at this scale, whereas only the flow velocity is relevant in this study. As stated in the methods section, only 2-sample t-tests were performed in this study between pairs of groups.

(For large datasets, or papers with a very large number of statistical tests, you may upload a single table file with tests, Ns, etc., with reference to sections in the manuscript.)

**Group allocation**

- Indicate how samples were allocated into experimental groups (in the case of clinical studies, please specify allocation to treatment method); if randomization was used, please also state if restricted randomization was applied
- Indicate if masking was used during group allocation, data collection and/or data analysis

Please outline where this information can be found within the submission (e.g., sections or figure legends), or explain why this information doesn’t apply to your submission:

For our studies, we assigned ablated embryos versus control siblings, or mutants versus control siblings. For the experiments on ciliary mutants, mutants were identified by their obvious phenotype (curled down) based on numerous publications and verification in our lab.

As stated in the methods section, for all manual measurements (central canal diameter, and measurement of growth in the embryo), the order of fish in the analysis pipeline was randomized so that the phenotype could not be inferred at any point during the data analysis.

**Additional data files (“source data”)**

- We encourage you to upload relevant additional data files, such as numerical data that are represented as a graph in a figure, or as a summary table
- Where provided, these should be in the most useful format, and they can be uploaded as “Source data” files linked to a main figure or table
- Include model definition files including the full list of parameters used
- Include code used for data analysis (e.g., R, MatLab)
- Avoid stating that data files are “available upon request”

Please indicate the figures or tables for which source data files have been provided:

Around 500Go of data were used to produce the figure of this article, so providing all raw files would be difficult. Nonetheless, when relevant, we have provided one raw test file, as well as the source code for analysis. Among others, we shared our code to generate the CSF flow profile. For all scatter plots, and histograms in the article, we have shared all the values (in .mat files) used to generate the plots, and sometimes have provided the code to plot this data accordingly to the article. Details on how to reproduce the plots and understand the organization of the data are provided within each folder. We also shared several .tif files where the different canals between the brain and the spinal cord can be captured.
